# Convergence of Innate and Adaptive Immunity during Human Aging

**DOI:** 10.3389/fimmu.2016.00445

**Published:** 2016-11-04

**Authors:** Branca I. Pereira, Arne N. Akbar

**Affiliations:** ^1^Division of Infection and Immunity, University College London, London, UK

**Keywords:** aging, immunosenescence, natural killer receptors, T cell receptor, innate-like T lymphocytes

## Abstract

Aging is associated with profound changes in the human immune system, a phenomenon referred to as immunosenescence. This complex immune remodeling affects the adaptive immune system and the CD8^+^ T cell compartment in particular, leading to the accumulation of terminally differentiated T cells, which can rapidly exert their effector functions at the expenses of a limited proliferative potential. In this review, we will discuss evidence suggesting that senescent αβCD8^+^ T cells acquire the hallmarks of innate-like T cells and use recently acquired NK cell receptors as an alternative mechanism to mediate rapid effector functions. These cells concomitantly lose expression of co-stimulatory receptors and exhibit decreased T cell receptor signaling, suggesting a functional shift away from antigen-specific activation. The convergence of innate and adaptive features in senescent T cells challenges the classic division between innate and adaptive immune systems. Innate-like T cells are particularly important for stress and tumor surveillance, and we propose a new role for these cells in aging, where the acquisition of innate-like functions may represent a beneficial adaptation to an increased burden of malignancy with age, although it may also pose a higher risk of autoimmune disorders.

## Introduction

Natural killer cells and αβCD8^+^ T lymphocytes are the two major cell lineages with constitutive cytotoxic activity and have a crucial role in the recognition and killing of abnormal cells. However, the paradigm for the recognition of target cells is fundamentally different between these two cell types: conventional αβCD8^+^ T cells rely on the T cell receptor (TCR) to recognize specific peptides presented by major histocompatibility complex class-I (MHC-I) molecules, whereas NK cells use a repertoire of germ line-encoded receptors to detect “missing self” or “altered-self” antigens and directly kill abnormal cells, without prior sensitization (1). Besides antigen specificity, the development of immunological memory is conventionally another distinctive feature between NK and T cells, categorizing them into distinct arms of the immune system and the innate and adaptive immune system, respectively (2).

Nevertheless, accumulating evidence supports the existence of NK cell memory (3, 4), as well as evidence for TCR-independent responses mediated by αβCD8^+^ T lymphocytes (5–7), suggesting that the conventional limits between the innate and adaptive arms of the immune system may be not as distinct as first thought (8). NK and T lymphocytes have a common origin from a lymphoid progenitor cell in the bone marrow (9), and recent comparative proteomic and transcriptomic studies have demonstrated a remarkably close proximity between effector αβCD8^+^ T lymphocytes and NK cells (10, 11), reiterating an evolutionary ancestry and shared biology between the two cell lineages.

An increasing body of literature reveals the existence of subsets of T cells with features that bridge innate and adaptive immunity (12–14). In humans, these innate-like T cells comprise the invariant natural killer T (iNKT) cells, CD1d-restricted natural killer T (NKT) cells, mucosa-associated invariant T (MAIT) cells, and γδT cells. These cells typically co-express a TCR and NK cell lineage markers, distinguishing them from NK cells and other innate lymphoid cells (ILCs), which lack the expression of a TCR or somatically rearranged receptors. Functionally, innate-like T cells respond to TCR ligation but are also able to respond rapidly to danger signals and pro-inflammatory cytokines, independently of TCR stimulation, resembling innate cells. Recently, subsets of conventional αβCD8^+^ T cells expressing NK cell markers and intraepithelial T cells have been included in this vaguely defined group of innate-like T cells (15, 16). Despite the similarities in phenotype and function, there are clear differences in ontogeny and tissue distribution between them.

In this review, we will discuss recent evidence that aging is associated with the expansion of a subset of conventional αβCD8^+^ T cells with phenotypic, functional, and transcriptomic features that resemble NK cells. Such innate-like αβCD8^+^ T cells have the characteristics of terminally differentiated T cells, and the acquisition of functional NK receptors is most likely part of a general reprograming of the CD8^+^ T cell compartment during human aging, to ensure broad and rapid effector functions. We propose that innate-like αβCD8^+^ T cells share important features with other innate-like T cells; however, fundamental differences in origin and development separate them from truly innate cells. Interestingly, these cells are also differentially affected by aging, suggesting distinct roles in immune responses at different times of life.

## Immunosenescence

Aging is associated with a general decline in immune function, contributing to a higher risk of infection, cancer, and autoimmune diseases in the elderly. Such faulty immune responses are the result of a profound remodeling of the immune system that occurs with age, generally termed as immunosenescence (17). While the number of naïve T cells emerging from the thymus progressively decreases with age as a result of thymic involution (18), the memory T cell pool expands and exhibits significant changes in the phenotype and function of antigen-experienced T cells, particularly evident in the CD8^+^ T cell compartment (19). Chronic immune activation due to persistent viral infections, such as cytomegalovirus (CMV) and Epstein–Barr virus (EBV), is one of the main drivers contributing to the accumulation of highly differentiated antigen-specific CD8^+^ T lymphocytes that have characteristics of replicative senescence (20, 21). In combination with the depletion of the peripheral pool of naïve T cells, the accumulation of these terminally differentiated T cells with age skews the immune repertoire and has been implicated in the impaired immune responses to new antigens and vaccination in the elderly (22, 23).

The widespread effects of aging on the immune system have been reviewed elsewhere (24) and include defects in the function of natural killer cells, neutrophils, macrophages, and dendritic cells as well as B cells and hematopoietic stem cells. In the innate immune compartment, changes in the phenotype and function of NK cells have been described (25) and associated with the accumulation of CD56^dim^ NK cells with a mature phenotype, characterized by the increased expression of maturation markers, such as CD57 (26) and KLRG1 (27, 28). Although the effects of aging on the cytolytic function of NK cells are still controversial, our group recently identified a subset of CD56^dim^ KLRG1^high^ NK cells that is expanded in the elderly, displaying impaired cytotoxicity and proliferation as well as other features of senescence (28).

While many aspects of the immune response are impaired, there is also evidence for hyperresponsiveness of the immune system during aging (29). It is likely that there is a complex remodeling of the immune system throughout life in an attempt to maintain effective immune responses, which could be beneficial in the responses to infections and cancer but may carry an increased risk of autoimmune and inflammatory diseases in the elderly (30).

## Characteristics of Highly Differentiated αβCD8^+^ T Cells

Multiple phenotypic and functional features have been proposed to define senescent CD8^+^ T cells (Table 1). Loss of co-stimulatory receptors, such as CD28 and CD27, is one of the most consistent immunological markers of T cell aging (31, 32) which, in combination with other markers of maturation such as CD45RA, KLRG1, and CD57 expression, identifies a subpopulation of long-lived immune cells with characteristics of terminal differentiation or senescence (33).

**Table 1:** **Phenotypic and functional characteristics of senescent CD8^+^ T cells, compared to less differentiated subsets**.

	Early differentiation	Intermediate differentiation	Terminal differentiation
**Phenotypic markers**			
CD28	++	+/−	–
CD27	++	+/−	–
CD45RA	++	+/−	+/−
CCR7	++	+	–
CD62L	++	+	–
CD57	–	+/−	++
KLRG1	–	+/−	++
Other NKR (KIR, NKG2, and CD56)	–	+/−	++
**Functional features**			
Proliferation	++	+	–
Telomerase activity	++	+	–
Telomeres	+++	++	+
Cytotoxicity	–	+	++
Cytokine secretion (TNF-α, IFN-γ)	–	+	++
**Signaling pathways**			
TCR signaling	+	++	+/−
IL-2 signaling	+	++	+/−
Pi3K–AKT–mTOR signaling	+	++	+/−
p38MAPK activation	–	–	+

*KLRG1, killer cell lectin-like receptor G1; NKR, natural killer receptor; KIR, killer cell immunoglobulin-like receptor; NKG2, natural killer receptor G2, TNF-α, tumor necrosis factor alpha; IFN-γ, interferon gamma; PI3K, phosphatidylinositol-3 kinase; mTOR, mammalian target of rapamycin*.

Several lines of evidence indicate that end-stage CD27^−^CD28^−^CD45RA^+^ CD57^+^ T cells accumulate significantly in older humans (34), during chronic viral infections (35) and in chronic inflammatory diseases (36). These cells exhibit the characteristic features of senescence that include accumulation of DNA damage markers, short telomeres, low proliferation, and loss in the capacity to activate the enzyme telomerase (37–39). A paradoxical observation is that senescent CD8^+^ T cells maintain potent effector functions, despite the loss of proliferative capacity, and thus should not be considered as a residual population of dysfunctional cells. On the contrary, these cells are polyfunctional, reflecting their ability to simultaneously carry out multiple functions, including secretion of IFN-γ and TNF-α and cytotoxicity (35, 38, 40), and this is an important observation that distinguishes senescent from exhausted T cells (41). Nevertheless, the increased secretion of pro-inflammatory cytokines by senescent T cells may have detrimental effects on the tissue microenvironment and contribute to the age-associated low-grade inflammatory state termed “inflammaging” (42).

Highly differentiated T cells have impaired TCR signaling (43, 44). We recently described that senescent CD27-28^−^ CD4^+^ T cells exhibit decreased expression of key components of the TCR signalosome, such as LCK, LAT, and SLP-76 (39), and found similar observations in end-stage CD8^+^ T cells. Interestingly, as T cells progressively differentiate, they concomitantly start expressing NK lineage receptors. Collectively, these observations suggest that, as CD8^+^ T cells terminally differentiate, they become less dependent on antigen-specific signals and more responsive to innate-like signals.

## TCR Hyporesponsiveness in Terminally Differentiated T Cells

Conventionally, optimal activation of T cells requires the engagement of the TCR and the second signal usually delivered by co-stimulatory receptors or cytokines. However, as previously mentioned, T cell senescence is associated not only with the loss of co-stimulatory receptors but also with impairment of TCR signaling (43, 44), leading to defects in classical T cell functions.

Changes in the composition of membrane lipids and lipid rafts have been described and linked to the age-related changes in TCR proximal signaling (45). More recently, Li and colleagues investigated the molecular mechanisms accounting for the loss of TCR sensitivity with age and found a correlation with the decreased expression of miR-181a and increased activity of DUSP6, a phosphatase that negatively regulates proximal TCR signaling (46). We recently demonstrated that the accumulation of DNA damage in senescent CD4^+^ T cells leads to the activation of AMP kinase, which is implicated in the decreased expression of key elements of the proximal TCR machinery, leading to impaired proximal TCR signaling in these cells (39). It is evident from these studies that aging is associated with a decrease in TCR responsiveness. Interestingly, recent studies have linked the acquisition of innate-like effector functions by memory CD8 T cells with defective TCR signaling (47–49).

## TCR-Independent Activation of αβCD8^+^ T Cells

Accumulating evidence indicates that memory CD8^+^ T cells may be activated in a TCR-independent manner through a process called bystander activation. This occurs in the absence of the cognate antigen, through the action of inflammatory cytokines, such as type I interferons (50, 51), IL-15 (52), IL-12 (53), IL-18, or a combination of these (5, 7, 54).

In addition to inflammatory cytokines, the acquisition of stimulatory innate immune receptors has been implicated in antigen-independent activation of CD8^+^ T cells. Among them, C-type lectin activating receptors, such as NKG2D and NKG2C, which recognize self-ligands related to the MHC-I have been shown to play crucial role in the mediation of innate-like responses by CD8^+^ T cells (6, 55). NKG2D is a classical example of a NK cell receptor that is highly expressed on αβCD8^+^ T cells and subsets of γδ T cells (56, 57). While the general consensus is that NKG2D engagement serves as a co-stimulatory receptor in CD8^+^ T cells, amplifying TCR signals in virus-specific responses (58) as well as antitumor immunity (59, 60), other studies have provided compelling evidence that CD8^+^ T cells may respond to NKG2D ligation alone, without TCR engagement, provided that cells are stimulated with cytokines, such as IL-15 or high doses of IL-2 (6, 49, 61, 62). Such TCR-independent, NKG2D-dependent mechanism of activation of CD8^+^ T cells has been shown important for host defense against infections (49) and tumor surveillance (63) but has also been implicated in the pathogenesis of inflammatory and autoimmune reactions (6).

Collectively, these findings challenge the classic paradigm that TCR engagement by the cognate antigen is necessary for the activation of T cells and support the role of innate-like receptors in the regulation of T cell effector functions. Overall, such observations may shed light on the question of how senescent T cells maintain potent effector functions, despite the TCR hyporesponsiveness.

## Expansion of αβCD8^+^ T Cells Expressing NK Cell Receptors with Aging

Studies in human centenarians have shown an increased proportion of T cells expressing NK cell receptors (NKRs), whereas these cells represent a minor population of circulating lymphocytes in newborns and young healthy individuals (64, 65). The frequency of NKR-expressing T cells not only increases with age but also in conditions associated with chronic immune activation (66–68). Among the most commonly observed NKR on T cells are activating and inhibitory receptors, such as CD16, CD56, CD57, NKp30, KLRG1, and CD94, members of the NK receptor G2 (NKG2), and killer-cell immunoglobulin-like receptor (KIR) families (10, 66–69).

Phenotypic analysis of NKR-expressing T cells revealed that the majority of these cells are highly differentiated effector memory CD8^+^ T cells, lacking CD28 expression and exhibiting other features of senescence (62, 69–71). Importantly, it has been demonstrated that these cells derive from conventional αβCD8^+^ T cells (71), express an oligoclonal αβTCR, and do not express the semi-invariant TCR Vα24/Vβ11 chains, excluding that they represent an expansion of the classical iNKT cells (17).

A recent study using single-cell mass cytometry to analyze the expression of NKR across the human immune system found, as expected, an increased expression of NK cell markers on CD8^+^ T cells, more evident in individuals with high levels of CD57, indicative of a terminally differentiated immune system (10). As the immune system matures, the diversity of the NKR repertoire increases on both NK and CD8^+^ T cells; however, the difference in magnitude for the gain of activating receptors appears to be much higher in CD8^+^ T than in NK cells. Hierarchical clustering based on NKR expression patterns unexpectedly clustered CD8^+^ T cells closer to mature NK cells than to CD4^+^ T cells.

Although the expansion of NKR-expressing T cells is mostly evident in the CD8^+^ T cell compartment, the expression of NK cell markers has also been found on human CD4^+^ T cells. For instance, our group and others have identified a subset of highly differentiated CD4^+^ T cells expressing NKG2D as well as cytotoxic granules, expanded in aging (35, 72) and autoimmune diseases (73, 74).

What triggers the expression of NKRs on T cells with aging is not yet clearly defined. TCR engagement and cytokine stimulation have been shown to induce the expression of NKRs on T cells both *in vitro* and *in vivo* (75, 76). In addition, studies in transplant recipients have demonstrated a striking upregulation of NKR in virus-specific CD8^+^ T cells after CMV reactivation (77), suggesting that chronic antigenic stimulation may drive the expansion of NKR-expressing T cells. Likewise, the upregulation of inhibitory NKRs, such as NKG2A and KLRG1, has been linked to clonal expansion after antigenic exposure and development of replicative senescence of T cells (20, 34, 78, 79).

## Reprograming of Senescent αβCD8^+^ T Cells into Innate-Like T Cells

The biological significance of NKR acquisition on CD8^+^ T cells during aging is not yet fully understood. It remains unclear whether the expansion of NKR-expressing T cells with age is a stochastic effect associated with chronic antigenic stimulation or whether it represents a predetermined program to allow these cells to respond rapidly in an innate-like fashion.

Functional studies performed with human CD8^+^ T cells that were activated and expanded *ex vivo* in the presence of cytokines or after TCR cross-linking, revealed that the acquisition of an NK cell phenotype was generally associated with the acquisition of functional features characteristic of NK cells (75). Of particular note, these cytokine-induced killer (CIK) cells develop the capacity to mediate MHC-unrestricted killing of target cells, in particular tumor cells, identifying them as potential tools in cancer therapy (80). Such activity does not require prior antigenic exposure but involves the engagement of stimulatory NKR and prior stimulation with inflammatory cytokines. Interestingly, these cells display a duality of function, as they are able to mediate both TCR-independent and antigen-specific immune responses (81).

Gene-expression studies have greatly contributed to dissecting the transcriptional changes occurring in aged T cells and shed light on the significance of NKR acquisition [reviewed in Ref. (82)]. Fann and colleagues originally compared the gene-expression profiles of human CD28^null^ and CD28^+^ memory CD8^+^ T cells and found significant changes in the CD28^null^ compartment, such as (1) decreased expression of co-stimulatory receptors, (2) acquired expression of NKRs (the majority of which have stimulatory activity), (3) upregulation of genes involved in cytotoxicity (in particular genes involved in the granule exocytosis pathway, perforin and granzymes, and in the Fas ligand/Fas pathway), (4) elevated expression of chemokines and cytokine receptors, and (5) differentially expressed signaling molecules and transcription factors (83). Subsequent studies comparing gene-expression profiles of CD8^+^ T cells between young and old donors have found similar changes, particularly in relation to enhanced expression of genes in the NK cell cluster (84, 85). Of particular note, Cao et al. described additional changes at the level of cell signaling pathways in aged CD8^+^ T cells, the most prominent involving an age-decreased expression of genes associated with TCR, IGF-1, and PI3K/AKT signaling pathways (85). Collectively, these studies point to a common transcriptional signature in aged CD8^+^ T cells that most likely reflect the acquisition of potent cytotoxic effector functions, largely independent of TCR signals.

It remains to be determined which transcriptional factors are the main regulators of this program. The differential expression of T-box transcription factors, T-bet and eomesodermin (Eomes) in aged T cells compared to the less differentiated counterparts (83), suggests a role in the reprograming of senescent CD8^+^ T cells. However, several other transcriptional regulators have been implicated in the terminal differentiation of cytotoxic CD8^+^ T cells, including the Foxo family of transcription factors [reviewed in Ref. (86), Blimp-1 (87, 88), ZEB2 (89), and promyelocytic leukemia zinc finger (PLZF) (90)]. Interestingly, some of these factors have been also implicated in the transcriptional control of NK and NKT cell differentiation (91, 92), and PLZF has been proposed as the key determinant factor for the development of innate T cells (92, 93). More importantly, overexpression of PLZF in conventional T cells was sufficient for the acquisition of innate-like phenotype and functions (90). Many questions remain in regard to the transcriptional program underlying T cell senescence. Importantly, it remains to be determined which factors control the peripheral modulation of TCR signaling and whether there is a mechanistic link between the acquisition of NKRs and the modulation of the TCR machinery.

**Figure 1:**
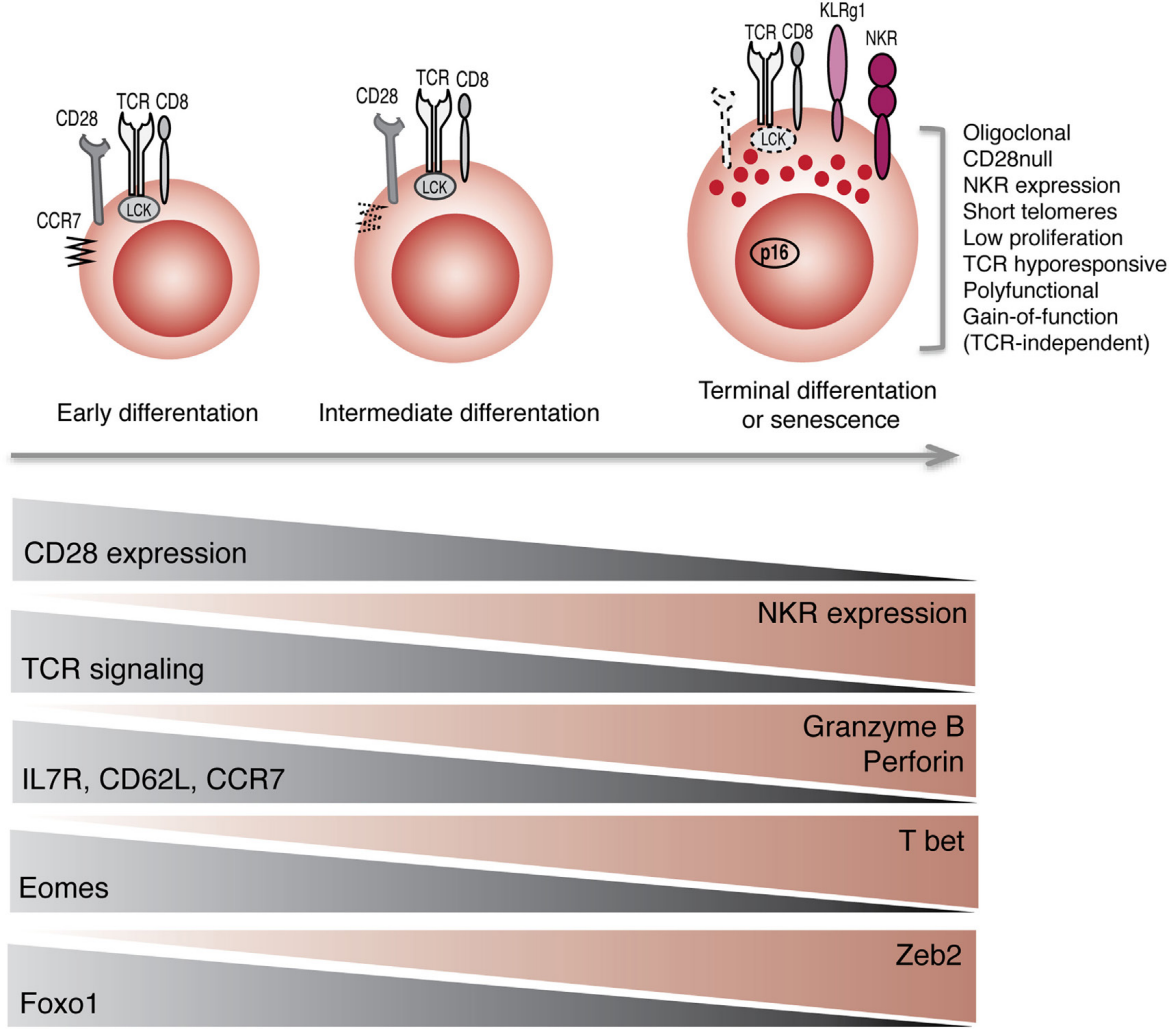
**Reprograming of CD8^+^ T cells into NK-like T cells with aging**. Progression toward terminal differentiation or senescence is associated with phenotypic, functional, and transcriptional changes that lead to the expansion of NK-like CD8^+^ T cells.

Collectively, these observations indicate that the acquisition of receptors that are normally found on NK cells may be part of a general reprograming of CD8^+^ T cells with maturation (Figure 1). Not only these cells acquire phenotypic markers of NK cells but also they acquire innate-like cytolytic functions, suggesting that a coordinated transcriptional program endows these cells with the machinery to respond to innate stimuli, without the requirement for TCR activation.

## Similarities and Differences with Innate-Like T Cells

Innate-like T cells are phenotypically characterized by the co-expression of a TCR with conventional NK cell lineage markers. NKT cells are the prototypical example of innate T lymphocytes. The term NKT cell is sometimes misused to refer to other subsets of conventional αβT cells that express NKRs, although there are fundamental differences between them. Classical NKT cells express an invariant TCR (Va24Ja18) that recognize glycolipids presented by the monomorphic CD1d molecule, and they account for 0.1–1% of T cells in human peripheral blood (94), whereas conventional αβT cells expressing NKR exhibit a diverse TCR repertoire and their frequency in peripheral blood is much higher, increasing with age and chronic inflammatory diseases (68). In striking contrast, aging is associated with decreased frequency and function of iNKT cells (95, 96).

Recently, it has been proposed that the suppression of TCR signaling is critical for the development of innate-like T cells. An elegant study done by Hayday and colleagues in mice models has brought some insights into how innate T cells downmodulate the TCR signaling machinery to allow an innate mode of activation in peripheral tissues, independent of the TCR (97). The authors demonstrated that this mechanism of TCR tuning after development in the thymus, concomitant with acquisition of responsiveness to innate signals is a feature shared by diverse subsets of innate-like T cells, including CD27^−^ γδT cells, mouse dendritic epidermal T cells (DETCs), and intestinal epidermal TCRαβ^+^ and γδ^+^ T cells. Although they could not find a similar mechanism to occur in iNKT cells, another study has demonstrated the acquisition of transient innate responsiveness by human iNKT cells *via* histone modifications induced by weak TCR stimulation (98).

Functionally, it has been demonstrated that innate-like lymphocytes are able to respond to TCR ligation as well as to innate signals alone, in particular to NKG2D ligation and to inflammatory cytokines (99–101). In humans, conventional αβCD8^+^ cells in celiac disease have been shown to respond to NKG2D ligands and pathological levels of IL-15, independently of TCR ligation (6). It remains to be determined if TCR signaling is suppressed in these cells, suggesting a common signature with other innate-like T cells.

Collectively, the observations that terminally differentiated CD8^+^ T cells co-express NKR and TCR have decreased TCR responsiveness and yet are able to respond rapidly to stimulation, without the requirement for cognate antigen supports the hypothesis that human senescent αβCD8^+^ T cells exhibit phenotypic and functional features that resemble other innate-like T cells. Nevertheless, the origin and development of human senescent αβCD8^+^ T cells is distinct from that of classical innate T cells. While innate T cells are developmentally pre-programed in the thymus (12), αβCD8^+^ T cells with innate-like features arise in the periphery, most likely as a result of a general reprograming driven by external environmental cues. The different origin may explain why aging is associated with a decreased frequency of innate T cells such as NKT cells, as a result of thymic involution, whereas the number of conventional αβT cells expressing NKR increases in the elderly, most likely a result of the homeostatic redistribution of T cells to compensate for the decrease in the output of T cells from the thymus with age.

## Physiological Role of Innate-Like αβCD8^+^ T Cells

The capacity to mediate dual innate and adaptive immune functions place senescent αβCD8^+^ T cells alongside other innate-like cells in the frontline of defense against pathogens and tumors. The acquisition of innate sensors specialized in the recognition of “danger” signals allows these cells to switch to a rapid and efficient mode of action in potentially harmful situations. Given the increased burden of tumors and infections with age, the contribution of such innate-like CD8^+^ T cells may be crucial. The capacity to mediate MHC-unrestricted killing against a broad array of tumor targets has been demonstrated *in vitro* and *in vivo* with CIK cells putting these cells as attractive candidates for immunotherapy in solid organ and hematopoietic cancer treatment (102). Interestingly, despite showing a decreased TCR responsiveness, studies indicate that these cells still retain the capacity to elicit specific TCR-dependent immune responses (81).

Nevertheless, the reversal of antigen-specific CD8^+^ T cells to an innate mode of function is not without consequence. The peripheral requirement for TCR engagement for T cell activation is an important control mechanism to prevent autoreactivity. In conditions associated with chronic activation and inflammation, the balance between activating and inhibitory signals may favor the onset of autoimmune reactions. Recent reports have demonstrated a role of NKG2D in CD8^+^ T cell activation in inflammatory states and other stress conditions where NKG2D ligands are induced in normal tissues, such as celiac disease (6), type I diabetes (103), and transplantation (104, 105).

## Concluding Remarks

In this review, we summarize evidence indicating that chronological aging is associated with accumulation of cells combining features of both the innate and adaptive arms of the immune system, most likely to compensate for functional defects of conventional NK and CD8^+^ T cells with age. We propose that senescent CD8^+^ T cells should not be seen as a dysfunctional population but instead a functionally distinct subset, which uses recently acquired NK cell machinery to maintain rapid effector functions throughout life. Contrary to the classic paradigm that peripheral TCR ligation is essential for T cell activation, this subset of highly differentiated T cells has impaired TCR responsiveness and may be non-specifically activated by inflammatory cytokines or after ligation of innate receptors. The switch to an innate mode of function may shed light on the mechanisms that allow highly differentiated CD8^+^ T cells to maintain they polyfunctionality, despite the loss of TCR signalosome.

Our understanding of the physiological significance of the expression of NKRs on T cells is still incomplete, and the identification of the molecular mechanisms and the transcriptional regulators underpinning the development of innate features in T cells is essential. Most importantly, it will be important to understand how the intersection between innate and adaptive immune features may be manipulated to enhance immune function and to use this information to develop new approaches to improve immunity in the elderly.

## Author Contributions

BP has done the literature search and writing. AA contributed for the writing and revising of the manuscript.

## Conflict of Interest Statement

The authors declare that the research was conducted in the absence of any commercial or financial relationships that could be construed as a potential conflict of interest.
